# Effects of the FIFA World Cup Qatar 2022 on match running performance in the Spanish professional soccer league: A pilot study

**DOI:** 10.5114/biolsport.2025.144414

**Published:** 2025-03-24

**Authors:** Tomás García-Calvo, José Carlos Ponce-Bordón, David Lobo-Triviño, Roberto López del Campo, Ricardo Resta, Javier Raya-González

**Affiliations:** 1 Faculty of Sport Sciences, University of Extremadura, Cáceres, Spain; 2 Department of Competitions and Mediacoach, LaLiga, Madrid, Spain; 3 Research Group on Sport and Physical Education for Personal and Social Development, Department of Specific Didactics, Faculty of Education Sciences and Psychology, University of Córdoba, Córdoba, Spain

**Keywords:** Football, High-speed running, International tournament, Match-analysis, Performance

## Abstract

The FIFA World Cup Qatar 2022 was scheduled during the in-season period of the European teams, impacting soccer clubs’ routines. This study aimed to analyse the influence of the FIFA World Cup Qatar 2022 on match running performance in the First Spanish soccer league, considering the players’ participation. A total of 11,530 individual match observations from 582 professional soccer players competing in the First Spanish soccer league (n = 370 matches) over the 2022/23 season were collected. Soccer players were classified according to participation in the World Cup: participants and non-participants. Pre-World Cup (1^st^–14^th^ match day) and post-World Cup (15^th^–38^th^ match day) periods were compared. Match running performance was obtained by the Mediacoach video tracking system in accordance with LaLiga. During the pre-World Cup period, no significant differences between groups were found, while in the post-World Cup period, non-participants covered significantly greater total distance, and distances at low, medium, and high speeds compared to the counterparts. No significant differences were observed between the periods for participants, although non-participant players covered a greater total distance, and distances at low, medium, high, and very high speeds, and sprinting during the post-World Cup period compared to the prior period. These results favour the inclusion of a long winter break in LaLiga to maintain or improve the physical performance of soccer players. Additionally, specific periodization of workload and recovery strategies for players participating in an international championship during the in-season period are crucial to prevent a decline of subsequent performance.

## INTRODUCTION

In recent years, the physical demands imposed on professional soccer players during official matches have significantly increased, specifically in terms of the distance covered at high intensity [1–3]. For instance, in a longitudinal study spanning four consecutive seasons in the Spanish LaLiga, Pons et al. [4] reported that Spanish professional soccer teams covered an average of 109,135 m per match, with distances of 22,436 m at 14–21 km · h^−1^, 3,019 m at 21–24 km · h^−1^, and 2,905 m at speeds above 24 km · h^−1^. As a result, the conditional dimension of training in professional soccer has become increasingly important. In this vein, enhancing the match running performance and reducing the injury risk have become the main concerns for strength and conditioning coaches working with professional soccer populations [5, 6, 7].

To implement appropriate training strategies, understanding the specific schedule of each country is essential [8]. In this regard, winter breaks applied in some federations seem to affect the players’ performance and injury incidence. For instance, Marques et al. [9] conducted a review and observed that break periods impaired physical performance in soccer players. However, these authors reported that the inclusion of specific training programmes during breaks could help to maintain or attenuate the deterioration of soccer player performance, justifying the inclusion of training camps during these periods of competitive inactivity. Regarding the impact of winter breaks on technical actions, Jamil et al. [10] found that winter breaks of less than 13 days would not affect technical performance levels in professional soccer players, while longer break periods (i.e., 19 to 32 days) impaired shooting and soccer performance. Analysing the influence of winter breaks on injury risk, Ekstrand et al. [11] compared injury rates among professional soccer teams with and without a winter break. The findings of this study showed that the absence of a scheduled winter break was associated with a higher injury burden in professional soccer teams. However, a recent study which retrospectively examined the effect of the 2022 FIFA World Cup (WC) on injury rates in French Ligue 1 soccer clubs showed that injury occurrence was substantially affected during the 2022/23 season when a mid-season WC was held with an increase of approximately 23% for the total number of injuries [12]. Therefore, these winter breaks must be considered in professional soccer teams’ periodization.

The FIFA WC is one of the most popular soccer events and is considered the main official tournament at the international level in this sport [13]. This tournament takes place every four years [14], generating great prestige for the winning national team. To date, the FIFA WC has always been played in the summer, after the official competitions have finished. However, in 2022, due to the weather in Qatar, this edition was held in December, during the in-season period of the European soccer season, leading to an unusual winter break in the Spanish LaLiga and representing a challenge for the staff of the participating national teams and the domestic clubs to which participating players are attached [15]. Some studies have tried to address this challenge through theoretical and experimental research. However, these studies did not involve real match situations, and most of the results were based on variables from the 2018 FIFA WC [16, 17]. A recent study compared the external load of non-participant soccer players in the FIFA WC 2022 in the eight matches before and after the WC, reporting enhanced performance in most external load metrics after the FIFA WC Qatar 2022 [18].

Nevertheless, since in LaLiga there are both international and non-international players, this winter break could affect them in a heterogeneous manner according to each player’s condition, increasing the match number for participants and allowing a rest period followed by a preparation phase for non-participants. Thus, it seems relevant to understand how the FIFA WC Qatar 2022 has impacted both player groups. Specifically, the aim of this study was to analyse the impact of the FIFA WC Qatar 2022 on match running performance in the First Spanish soccer league, differentiating between players who participated in the WC and those who did not. It was hypothesized that players participating in the FIFA WC Qatar 2022 could experience a reduction in the physical performance after the tournament due to the fatigue generated by the accumulation of official matches.

## MATERIALS AND METHODS

### Study Design

A longitudinal, retrospective, and quasi-experimental design was employed to compare changes in match running performance in the Spanish professional soccer league after the FIFA WC Qatar 2022. Two different periods of the 2022/23 season were considered: the first period (i.e., pre-WC; *n* = 4,364 observations) encompassed matchdays 1 to 14, and the second period (i.e., post-WC; *n* = 7,166 observations) spanned matchdays 15 to 38. Furthermore, to examine the differences in match running performance after the FIFA WC Qatar 2022 according to different types of players, the participation of soccer players in the WC was considered. This variable was included as a quantitative variable: participants (i.e., players who competed in the FIFA WC Qatar 2022 tournament; *n* = 2,266 observations), and non-participants (i.e., players who did not compete in the FIFA WC Qatar 2022 tournament; *n* = 9,624 observations).

### Participants

The sample comprised 11,530 individual match observations from 582 professional soccer players who competed in the First Spanish soccer league (*n* = 370 matches) over the 2022/23 season. Players were split according to the participation in the WC: participants (*n* = 83 players), and non-participants (*n* = 499 players). All players who participated in matches (i.e., starters and non-starters) were included, except for players who competed for less than 10 minutes during the matches, as it was observed that the average values obtained from these players were higher than the team average [19]. Goalkeepers were not included in the analysis due to the specific role during the game. Data were retrieved from the Spanish Professional Soccer League (i.e., LaLiga), which allowed the use of the variables included in this investigation. In accordance with the ethical guidelines of LaLiga, this investigation does not include information that identifies soccer players (General Assembly of LaLiga, 2019). The study received full approval from the Bioethics Committee of the University of Extremadura (application number 239/2019).

**FIG. 1:**
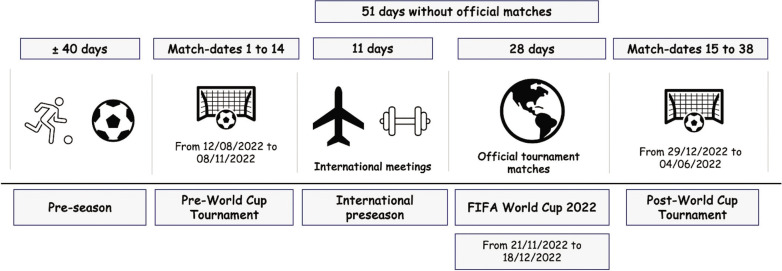
Official competition schedule during 2022/23 season for the First Spanish soccer league.

### Procedures and Variables

Match running performance data were obtained using the TRACAB multicamera computerized optical tracking system (ChryronHego VID, New York, NY) managed through the Mediacoach application (LaLiga, Madrid, Spain), with a sampling frequency of 25 Hz. The validity and reliability of this system for the variables used have been previously investigated [20, 21], demonstrating strong correlations (*r* > 0.80) and high intraclass correlation coefficients (*r* > 0.75) between the Mediacoach multicamera tracking system and the Global Positioning System (GPS). Additionally, small (< 0.30) to moderate (> .60) standard errors of estimate were observed in all speed categories used in this study. Match running performance was divided into the following categories: total distance covered by soccer players in metres (TD), low-speed running (LSR, distance covered at 7.2–14.4 km · h^−1^), medium-speed running (MSR, distance covered at 14.4–19.8 km·h^−1^), high-speed running (HSR, distance covered at 19.8–25.2 km · h^−1^), very high-speed running (VHSR, distance covered above 25.2 km·h^-1^), and sprinting speed running distance (Sprint, distance covered above 28 km · h^−1^). Absolute values of match physical demands variables were normalized to relative values per unit of time (i.e., m/min) to account for possible differences in the total playing time of soccer players. All efforts that involved a minimum movement of one metre, maintained for at least one second, were recorded.

### Statistical Analysis

All statistical analyses were performed using RStudio [22]. Considering the characteristics of the sample, which are organized hierarchically and nested in groups, and given the longitudinal structure of the data, linear mixed models (LMM) were determined as the best procedure for analysing the data. LMM have demonstrated the ability to handle unbalanced and repeated-measures data [23]. For example, match running performance variables in matches are nested within players (i.e., each player has a record for every match they have participated in, and each match has observations for several players). Players, in turn, are also nested within different teams each season. Thus, cross-classified multilevel models are suitable for data structures that are not purely hierarchical. Consequently, a general multilevel modelling strategy was applied, where fixed and random effects were included in different steps [23]. Therefore, LMM were used to analyse the influence of the FIFA WC Qatar 2022 on match running performance. Firstly, different models were performed for each of the dependent variables (i.e., distances covered at different speed thresholds), with the different phases of the competition (i.e., pre- and post-WC periods) and the different types of playing participation (i.e., participants and non-participants) included as fixed effects. The soccer player variable was considered as the random effect in the analysis. Following the procedure proposed by Heck & Thomas [23], models with different random effects (i.e., intercepts and slopes) were created for each variable. The difference between both non-participant and participant players before and after the FIFA WC Qatar 2022 was analysed by the interaction. Values were represented as coefficients and standard errors (Coeff ± SE). Statistical significance was set at *p* < 0.05. Finally, Cohen’s effect sizes (ES) were also calculated to quantify the magnitude of the difference for all pairwise comparisons using the following thresholds for interpretation: trivial, < 0.20; small, 0.20–0.59; moderate, 0.60–1.19; large, 1.20–1.99; and very large, > 2.00 [24].

## RESULTS

Table 1 presents match running performance variables categorized by season periods (i.e., pre-, and post-WC) and players’ participation in the FIFA WC Qatar 2022. Regarding players’ participation, during the pre-WC period, no significant differences were observed between player groups. In the post-WC period, non-participants covered significantly greater TD (*p* < .01), LSR (*p* < .05), MSR (*p* < .01), and HSR (*p* < .01) distances than participants. Concerning the periods, non-participants covered significantly greater TD (*p* < .001), LSR (*p* < .01), MSR (*p* < .001), HSR (*p* < .001), VHSR (*p* < .001), and Sprint distance (*p* < .05) during the post-WC period compared to the pre-WC period. No significant differences were found between the periods for the participant group.

**TABLE 1:** Differences in match running performance according to periods and players’ participation in FIFA World Cup Qatar 2022

		Pre-World Cup			Post-World Cup			Between-group differences	*p*	*d* (CI_95%_)	Interaction Effect *p*-value

		Coeff (SE)	*p*	*d* (CI_95%_)	Coeff (SE)	*p*	*d* (CI_95%_)				
TD (m × min.)	Non-participants	114.12 (.466)	.151	.16 (.09, .24)	115.15 (.458)	.004	.23 (.17, .29)	1.03	< .001	-.06 (-.10, -.02)	< .001
		
	Participants	112.38 (1.114)			111.72 (1.103)			-.66	.073	.01 (-.07, .09)	

LSR (m × min.)	Non-participants	44.21 (.288)	.173	.17 (.09, .24)	44.53 (.283)	.015	.23 (.17, .29)	.32	.010	.-.03 (-.07, .01)	.004
		
	Participants	43.20 (.685)			42.74 (.677)			-.46	.060	.03 (-.05, .11)	

MSR (m × min.)	Non-participants	20.15 (.235)	.063	.20 (.12, .27)	20.64 (.232)	.002	.28 (.22, .34)	.49	< .001	-.07 (-.12, -.03)	< .001
		
	Participants	19.01 (.565)			18.82 (.561)			-.19	.258	.01 (-.07, .10)	

HSR (m × min.)	Non-participants	7.87 (.110)	.054	.15 (.08, .23)	8.13 (.108)	.009	.20 (.14, .26)	.26	< .001	-.09 (-.13, -.05)	.098
		
	Participants	7.32 (.261)			7.40 (.258)			.08	.427	-.04 (-.13, .04)	

VHSR (m × min.)	Non-participants	2.26 (.056)	.561	-.05 (-.12, .03)	2.36 (.055)	.869	.01 (-.05, .06)	.10	< .001	-.07 (-.11, -.03)	< .097
		
	Participants	2.34 (.133)			2.33 (.130)			-.01	.881	-.02 (-.10, .07)	

Sprint (m × min.)	Non-participants	.79 (.029)	.363	-.08 (-.16, -.01)	.82 (.028)	.530	-.08 (-.14, -.02)	.03	.045	-.03 (-.08, .01)	.546
		
	Participants	.86 (.067)			.87 (.066)			.01	.733	-.03 (-.12, .05)	

*Note*: Coeff = Coefficient; SE = Standard Error; CI = Confidence Intervals; m × min. = meters per minute; TD = total distance covered by soccer players; LSR = distance covered at 7.2 to 14.4 km · h^−1^; MSR = distance covered at 14.4 to 19.8 km · h^−1^; HSR = distance covered at 19.8 to 25.2 km · h^−1^; VHSR = distance covered above 25.2 km · h^−1^; Sprint = distance covered above 28 km · h^−1^.

## DISCUSSION

This study aimed to investigate the impact of the FIFA WC Qatar 2022 on match running performance in the First Spanish soccer league, differentiating between participants and non-participants. As this is the first time that a FIFA WC has been held during the in-season period rather than after it, there are currently no studies that have analysed the effects of this change in the temporary location of the competition on the match running performance of the teams considering the players’ participation in this tournament. The main findings of the study were that: a) during the period prior to the WC, no significant differences between non-participants and participants were found; b) in the post-WC period, non-participants covered significantly greater distances at TD, LSR, MSR and HSR compared to the counterparts; c) no significant differences were observed between the periods for the group of participants, although non-participant players covered greater distances at TD, LSR, MSR, HSR, VHSR and Sprint during the post-WC period compared to the prior period.

In order to establish training tasks adjusted to each player’s characteristics, it is necessary to consider different variables such as age, competitive level, playing style, playing or match status, ball possession or team formation, as these factors could influence the external demands [25, 26, 27]. In this regard, the status of being an international player (i.e., players selected by the national teams) could be one of these factors, especially when international tournaments such as the WC are approaching, and players may need to self-manage the physical efforts [15, 16]. In our study, no significant between-group differences were observed in the pre-WC period. Interestingly, non-participant players covered a greater TD and distances at medium to high intensity, while participant players completed greater distances at very high intensity and sprinting. This could be related to the profiles sought by national staffs, with high-standard players being those with the highest physical fitness levels in the variables that determine soccer performance [28]. Conversely, during the post-WC period, significant between-group differences were found in TD, LSR, MSR, and HSR in favour of non-participant players. This could be due to the impact of increasing the number of matches within a congested calendar, which is more relevant in Spain, where a large proportion of the players whose teams reach the final rounds of the WC compete in LaLiga [29]. Also, these differences could be related to the specific preparation that non-participant players performed during the break due to the WC, facilitating adequate training periodization due to the absence of official matches [9]. Therefore, it seems necessary that in similar situations, the condition of each player (i.e., participant or non-participant) should be considered to individualize the periodization of the pre- and post-WC season in terms of workload and recovery, so that the international competition does not impact the performance of the teams.

Despite the intra-group differences observed, mainly in the post-WC period, it is worth noting the intra-group effects of including an unusual winter break for the group of non-participants, as well as the increase in the number of matches in a high-level world competition for the participants. In this regard, non-participant players increased the distance covered at all intensities after the WC, while participants covered shorter distances at TD, LSR, MSR, and VHSR after the WC compared to the prior values. Also, significant differences were observed in the effect of the WC in TD, LSR and MSR in favour of the group of non-participating players. Similar results for non-participant players were obtained by Reverte-Pagola et al. [18], who reported that external load parameters were greater after the FIFA WC Qatar 2022. Our findings suggest that the winter break has a stronger influence on the improvement of physical performance than the increase in the number of matches during the WC, in terms of fatigue and subsequent performance reduction [9]. Thus, perhaps it would be an appropriate strategy to include a winter break in LaLiga, not only to increase the performance of the footballers, but also to reduce the risk of injury [11]. Curiously, participant players increased (though not significantly) the distance covered at HSR and sprinting, indicating that the performance level of high-quality standard players influences the competition, especially when they are competing for a title with the teams.

Despite the promising results obtained, this study has some limitations that practitioners should take into account. Firstly, only players from one league (i.e., LaLiga) participated, where a winter break is not implemented, and most participants are members of top national teams, so these analyses should be performed in different European soccer leagues to extrapolate the results and ensure greater representativeness of this issue. Secondly, other contextual-related variables that could influence match running performance, such as age [19], competitive level [30], playing style [31], playing status or scoreline [26, 32], and team formation [25], were not considered. In addition, tactical-technical variables should be considered in future studies to perform a holistic analysis of the influence of WC on soccer performance. Thirdly, further studies should consider the type of player participation of soccer players in the domestic clubs and national teams (starter, non-starter, minutes played, etc.), since research has suggested that non-starters could modify the player activity profile in the last minutes of the matches [33]. In addition, the different types of player participation could lead to different solutions on how to manage the challenges related to the external load monitoring of soccer players [15]. Considering this issue, effective playing time should be considered in future to achieve a comprehensive analysis of the real physical demands of official soccer matches [34]. Finally, only male professional soccer players were involved, so it could be interesting to replicate this study in the Female Spanish professional soccer league to confirm the findings and compare results.

## CONCLUSIONS

In conclusion, this is the first study to date that has examined the influence of the FIFA WC Qatar 2022 on match running performance in a European soccer league, considering the players’ participation. The main results demonstrated that during the post-WC period, non-participants covered significantly greater distances at TD, LSR, MSR and HSR compared to participants. In addition, no significant differences were observed between the periods for the group of participants, although non-participant players covered greater distances at TD, LSR, MSR, HSR, VHSR and Sprint in the post-WC period compared to the prior period. These results suggest that incorporating a long (e.g., 10 to 19 days off) winter break with active recovery work at the beginning, reconditioning training, and tapering at the end, could be a strategy to maintain or even improve the physical performance of soccer players in LaLiga. Additionally, implementing specific periodization of workload and recovery strategies after the international participation for players participating in an international championship during the in-season period is crucial to prevent a decline in subsequent performance.

## Data Availability

Restrictions apply to the availability of these data. Data were obtained from LaLiga and are available at https://www.laliga.es/en with the permission of LaLiga.
